# Supplementary material for: Structural and Mechanistic Basis for the Inactivation of Human Ornithine Aminotransferase by (3S,4S)-3-Amino-4-fluorocyclopentenecarboxylic Acid
Source: Molecules. 2023 Jan 23;28(3):1133. doi: 10.3390/molecules28031133 (PMC9921285; doi:10.3390/molecules28031133)
Supplement: Supplementary file 1 [file molecules-28-01133-s001.zip › molecules-2140451-supplementary.pdf]

Supplementary Materials

Structural and Mechanistic Basis for the Inactivation of Human Ornithine Aminotransferase by (*S*)-3-Amino-(*S*)-4-fluorocyclopentenecarboxylic Acid

Sida Shen[1,†], Arseniy Butrin[2,†], Brett A. Beaupre[2], Glaucio M. Ferreira[4], Peter F. Doubleday[3,‡], Daniel H. Grass[1], Wei Zhu[1], Neil L. Kelleher[1,3], Graham R. Moran[2], Dali Liu[,2], and Richard B. Silverman*[,1,3,5]*

[1]Department of Chemistry and Center for Developmental Therapeutics, Northwestern University, Evanston, Illinois 60208, United States

[2]Department of Chemistry and Biochemistry, Loyola University Chicago, Chicago, Illinois 60660, United States

[3]Department of Molecular Biosciences, Northwestern University, Evanston, Illinois 60208, United States

[4]Department of Clinical and Toxicological Analyses, School of Pharmaceutical Sciences, University of São Paulo, São Paulo, SP, 05508-000, Brazil

[5]Department of Pharmacology, Northwestern University, Chicago, Illinois, 60611, United States

[†]These authors contributed equally.

Corresponding authors:

*(R.B.S.) E-mail: r-silverman@northwestern.edu. Phone: +1-847-491-5653;

*(D.L.) E-mail: dliu@luc.edu. Phone: +1-773-508-3093.

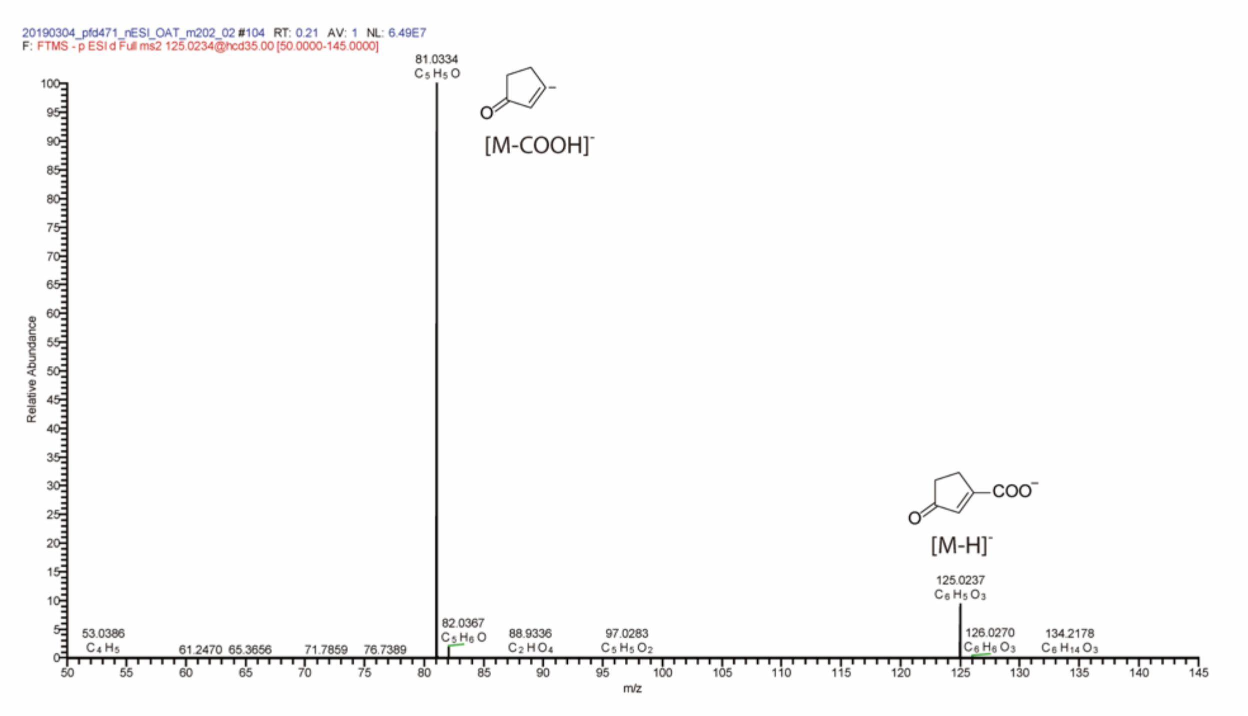

Figure S1. Primary metabolite **Met II** of **5** in *h*OAT (see Scheme 7)

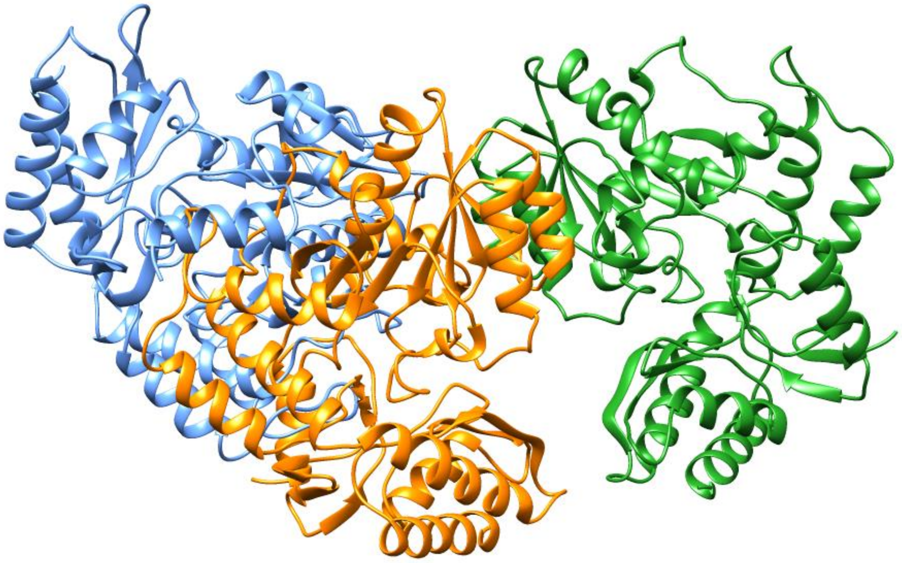

Figure S2. The asymmetric unit of the co-crystal structure of *h*OAT inactivated by **5**. The model contains three protein monomers, each monomer is shown in a different color.

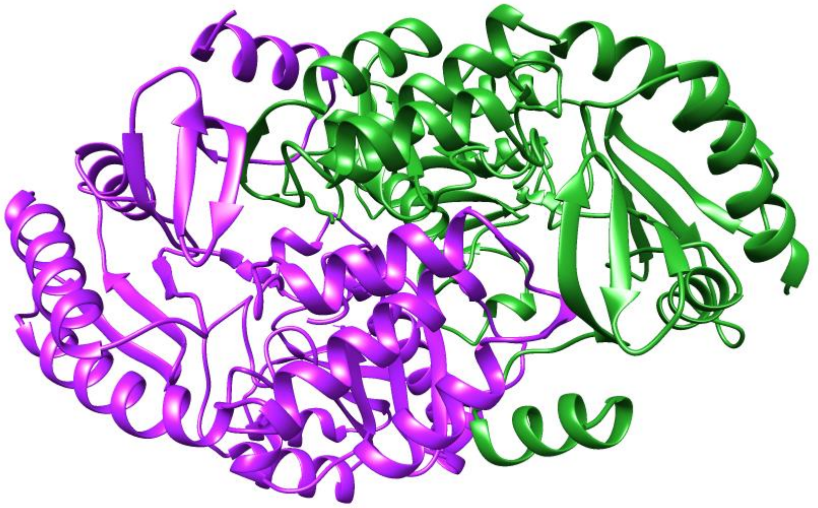

Figure S3. The biological assembly of *h*OAT represents a homodimer.

Table S1. Statistics of the crystal structures of *h*OAT inactivated by **5**

PDB code	8EZ1
Space group	C121
**Cell dimension**	
α, β, γ (deg)	90, 103.7, 90
a, b, c (Å)	202.2, 110.3, 57.1
Processed Resolution (Å)	1.91
Rmerge [b] (%)	18.5 (121.4)
Rpim [d] (%)	9.9 (67.3)
I/σ (I)	6.1 (1.1)
$CC_{1/2}$ [e] (%)	99.2 (46.4)
Completeness (%)	98.0 (95.5)
Multiplicity	4.2 (4.1)
No. Reflections	391615
No. Unique Reflections	92197
**Refinement**	
Rwork [f] / Rfree [g] (%)	18.85/23.66
No. of Atoms	
protein	9425
ligand	81
water	707
B factors ($Å^2$)	
protein	37.00
Ligand (copy A)	25.48
Ligand (copy B)	29.94
Ligand (copy C)	41.72
RMSD [h]	
bond lengths (Å)	0.012
bond angles (deg)	1.12
**Ramachandran plot (%)**	

favored	95.07
allowed	4.59
outliers	0.33

[a]Provided Resolution at $I/\sigma = 2$ for conventional assessment of data quality

[b]$R_{merge} = \Sigma|I_{obs} - I_{avg}|/\Sigma I_{avg}$

[c]The values for the highest-resolution bin are in parentheses

[d]Precision-indicating merging R

[e]Pearson correlation coefficient of two "half" data sets

[f]$R_{work} = \Sigma|F_{obs} - F_{calc}|/\Sigma F_{obs}$

[g]Five percent of the reflection data were selected at random as a test set, and only these data were used to calculate R_{free}

[h]Root-mean square deviation
